# Tamoxifen transiently disrupts estrous cyclicity without altering long-term ovarian aging trajectories

**DOI:** 10.64898/2026.09.03.749251

**Published:** 2026-09-08

**Authors:** Sarai Badillo, Ewa Poljanska, Sarah Ray, Hana Kilani, Jillian E.J. Cox, Sunghwan Ko, Subhasri Biswas, Doris M. Benbrook, Michael B. Stout, Sarah R. Ocañas

## Abstract

The ovary is both one of the earliest organs to functionally age in the body, with declines emerging well before reproductive failure and contributing to systemic aging. Since ovarian function depends on tightly regulated hormonal and inflammatory cycles, even subtle disruptions can confound aging-related phenotypes. Tamoxifen-inducible Cre systems are widely used to study ovarian biology, however, tamoxifen is a selective estrogen receptor modulator capable of disturbing ovarian physiology. This introduces a critical and often overlooked concern that tamoxifen-based models may produce lasting effects that obscure true biological signals, particularly in ovarian aging studies. To address this, we tested the hypothesis that tamoxifen induces transient physiological disruption without altering long-term transcriptional outcomes. Female mice were treated with tamoxifen or vehicle at 3 months of age, and estrous cyclicity was monitored longitudinally by vaginal cytology at 3 days, 2 months, 3 months, 6 months, and 12 months post-induction. Ovaries were collected at young (6 months) and aged (12 months) time points for bulk RNA sequencing, followed by differential gene expression and pathway-level analyses. We found that tamoxifen treatment disrupted estrous cyclicity shortly after administration, confirming short term physiological effects. However, normal cycling was restored by 3 months post-treatment, indicating recovery of ovarian function. At the transcriptional level, minimal differences were observed between tamoxifen and vehicle treated groups at both young and aged time points. In contrast, aging-associated transcriptional programs were conserved across treatment conditions with shared alterations in pathways related to extracellular matrix remodeling, senescence, and cellular homeostasis. Together these findings demonstrated that while tamoxifen disturbs ovarian physiology in the short term, it does not produce lasting transcriptional effects or aging associated phenotypes. These results resolve a critical methodological concern and provide validation for the use of tamoxifen-inducible systems in ovarian research, supporting their application in studies of reproductive aging and systemic female health when proper controls are integrated into study design.

## Full Text Availability

The license terms selected by the author(s) for this preprint version do not permit archiving in PMC. The full text is available from the preprint server.

